# Isotemporal substitution of sedentary time with physical activity and sleeping time: associations with body composition among individuals with prediabetes

**DOI:** 10.3389/fspor.2025.1579962

**Published:** 2025-04-07

**Authors:** M. Torres-Carballo, A. M. Galmes-Panades, M. Arias-Fernández, A. Huguet-Torres, M. Abbate, S. Fresneda, C. Sánchez-Rodríguez, A. M. Yañez, M. Bennasar-Veny

**Affiliations:** ^1^ Research Group on Global Health, University of Balearic Islands, Palma, Spain; ^2^ Primary Care of Mallorca, Public Health Service of the Balearic Islands, Palma, Spain; ^3^ Research Group on Nursing, Community & Global Health, Health Research Institute of the Balearic Islands (IdISBa), Palma, Spain; ^4^ CIBER of Physiopathology of Obesity and Nutrition (CIBEROBN), Instituto de Salud Carlos III, Madrid, Spain; ^5^ Physical Activity and Sport Sciences Research Group (GICAFE), Institute for Educational Research and Innovation, University of the Balearic Islands, Palma, Spain; ^6^ Nursing and Physiotherapy Department, University of the Balearic Islands, Palma, Spain; ^7^ Hospital Sant Joan de Deu, Palma, Spain; ^8^ Network for Research on Chronicity, Primary Care, and Health Promotion (RICAPPS), Palma, Spain; ^9^ Centre for Biomedical Research Network (CIBER) in Epidemiology and Public Health (CIBERESP), Madrid, Spain

**Keywords:** body composition, visceral adipose tissue, physical activity, sedentary time, obesity, prediabetes

## Abstract

**Aim:**

To assess the association between physical activity (PA), sedentary time (ST), and sleep with body composition, and to explore the effects of reallocating ST to PA or sleep on body composition in individuals with prediabetes and overweight/obesity.

**Material, methods and results:**

Baseline data from the PREDIPHONE trial, including 159 participants (mean age 59.6 years) with prediabetes (Fasting Plasma Glucose 100–125 mg/dl) and overweight/obesity (Body Mass Index 27–40 kg/m²), were analyzed. Body composition was assessed via bioelectrical impedance, while PA, ST, and sleep were measured with accelerometry. Linear regression and isotemporal substitution models evaluated associations. Increased ST was positively associated with body fat mass (kg) (*β* = 0.016; CI 95%: 0.003–0.030), body fat mass (%) (*β* = 0.009; 0.001–0.018), and visceral adipose tissue (*β* = 0.005; 0.001–0.010). Moderate-to-vigorous PA (MVPA) was negatively associated with body fat mass (kg) [*β* = −0.031; 0.055- (−0.008)], body fat mass (%) [*β* = −0.017; −0.032-(−0.003)], and Visceral adipose tissue [*β* = −0.009; −0.02-(−0.002)]. Replacing ST with MVPA was linked to lower Visceral adipose tissue [*β* = −0.012; −0.024-(−0.001)] and body fat mass (kg) [*β* = −0.039; −0.074-(−0.006)], but not with lean mass. No significant associations were found when substituting ST with light PA or sleep.

**Discussion:**

In individuals with prediabetes and overweight/obesity, replacing ST with MVPA could reduce body fat and VAT but not increases lean mass.

## Introduction

Obesity is a multifactorial, chronic, and complex condition characterized by adipose tissue expansion, which significantly increases the risk of cardiometabolic diseases such as type 2 diabetes (T2D) and cardiovascular disease (CVD) (1–3). Obesity represents a serious public health concern due to its rising global prevalence (3). In 2020, 14% of the population worldwide suffered from obesity, and this prevalence is predicted to rise to 24% by 2035 (2). In Spain, the prevalence of obesity in 2022 was 22%, or rather well above the worldwide average (4). Importantly, this prevalence is also rising among middle-aged and older individuals, possibly accelerating the likelihood of new-onset cardiometabolic conditions in these populations (5).

The age-related development of obesity is likely associated with a state of positive energy balance, dependent on lifestyle changes characterized by reduced physical activity (PA), increased sedentary behavior and increased caloric intake (5). Moreover, ageing leads to a series of changes in body composition, including an increase of adipose tissue, redistribution of fat, increasing fat accumulation in the visceral area, and accumulation in ectopic areas such as skeletal muscle and liver (6–9). However, these changes can be followed by loss of muscle mass, reduced strength, and decreased function, frequently progressing to sarcopenic obesity (10). Such obesity often results in functional limitations in the short term, and lower capacity to perform PA in the long term (10). These physiological changes significantly affect glucose metabolism and insulin sensitivity, and stimulate inflammatory processes, thereby accelerating the development of chronic conditions such as T2D and CVD (11–13). Notably, with ageing, visceral adipose tissue promotes systemic insulin resistance and chronic inflammation even when Body Mass Index (BMI) is in the normal or overweight categories and is an independent predictor of diabetes risk and excess mortality (11, 12, 14).

In individuals with overweight or obesity, a 5%–10% weight loss is considered clinically significant in preventing or delaying the onset of chronic conditions such as T2D and CVD (15, 16). However, if weight loss strategies such as caloric restriction are not accompanied by PA, they may lead to loss of lean mass, further exacerbating the decline in muscle quality and strength seen in age-related sarcopenic obesity (7, 17). Reducing sedentary time (ST) and increasing regular PA over the long term are, in fact, key strategies that preserve muscle mass while addressing obesity, and should always be recommended (18, 19). It has been observed that a combination of aerobic and resistance training effectively improves body composition [preserves muscle mass and reduces Visceral adipose tissue *(*VAT) and ectopic fat], physical function, and cardiometabolic health (reduces skeletal inflammatory markers and ameliorates insulin resistance and pancreatic β-cell function, aiding glucose homeostasis) (20–24), besides improving mental health and overall well-being (19).

Given these considerations, weight loss recommendations for middle-aged and older individuals should not be based solely on body weight or BMI but rather on body composition and other anthropometric measurements such as waist circumference (WC) and take into account not only the absolute values of each component but also their distribution in the body (25, 26).

Isotemporal substitution models are recognized as one of the most suitable statistical approaches for examining the relationships between reallocating activity patterns—particularly time spent in sedentary behavior and physical activity—and health outcomes. This analytical method acknowledges the finite nature of time (27, 28), meaning that an increase in one behavior (e.g., sedentary time) inevitably reduces time allocated to another (e.g., moderate-to-vigorous physical activity). Furthermore, it considers the interdependence of daily behaviours, including sleep, sedentary time, and physical activity (28, 29). By applying this model, valuable insights can be gained into how changes in activity distribution affect cardiometabolic markers in adults. This is essential for designing tailored interventions aimed at enhancing body composition in this population.

For all of the above, the objectives of this cross-sectional analysis were to (1) assess whether PA, ST and sleeping time are associated with body composition, and (2) explore whether reallocating ST to PA at different intensities or sleeping time is associated with differences in body composition in individuals with overweight/obesity and at increased T2D risk.

## Material and methods

### Study design

The present cross-sectional analysis includes baseline data from the PREDIPHONE trial (clinicaltrial.gov identifier: NCT04735640 registered on February 3, 2021), which assesses the effectiveness of a nurse-led personalized telephone intervention on lifestyle changes in people with prediabetes. The design and methods of the PREDIPHONE trial have been previously described (30). The study was approved by the Institutional Review Board of the Research Ethics Committee of the Balearic Islands (CEI-IB Ref No: IB 3947/19 PI) and complies with the Declaration of Helsinki ethical standards. Informed consent was obtained from all subjects involved in the study.

### Study sample

Between May 2021 and September 2022, a total of 206 participants were screened and recruited in 5 primary care centers of Palma, Mallorca, Spain. Inclusion criteria were age 25–75 years, BMI 27–40 kg/m^2^, Fasting plasma glucose 100–125 mg/dl (American Diabetes Association criteria) (31), none use of oral antidiabetic medication, and written informed consent. Further details of inclusion/exclusion criteria are reported in the published protocol (30).

**Figure 1:**
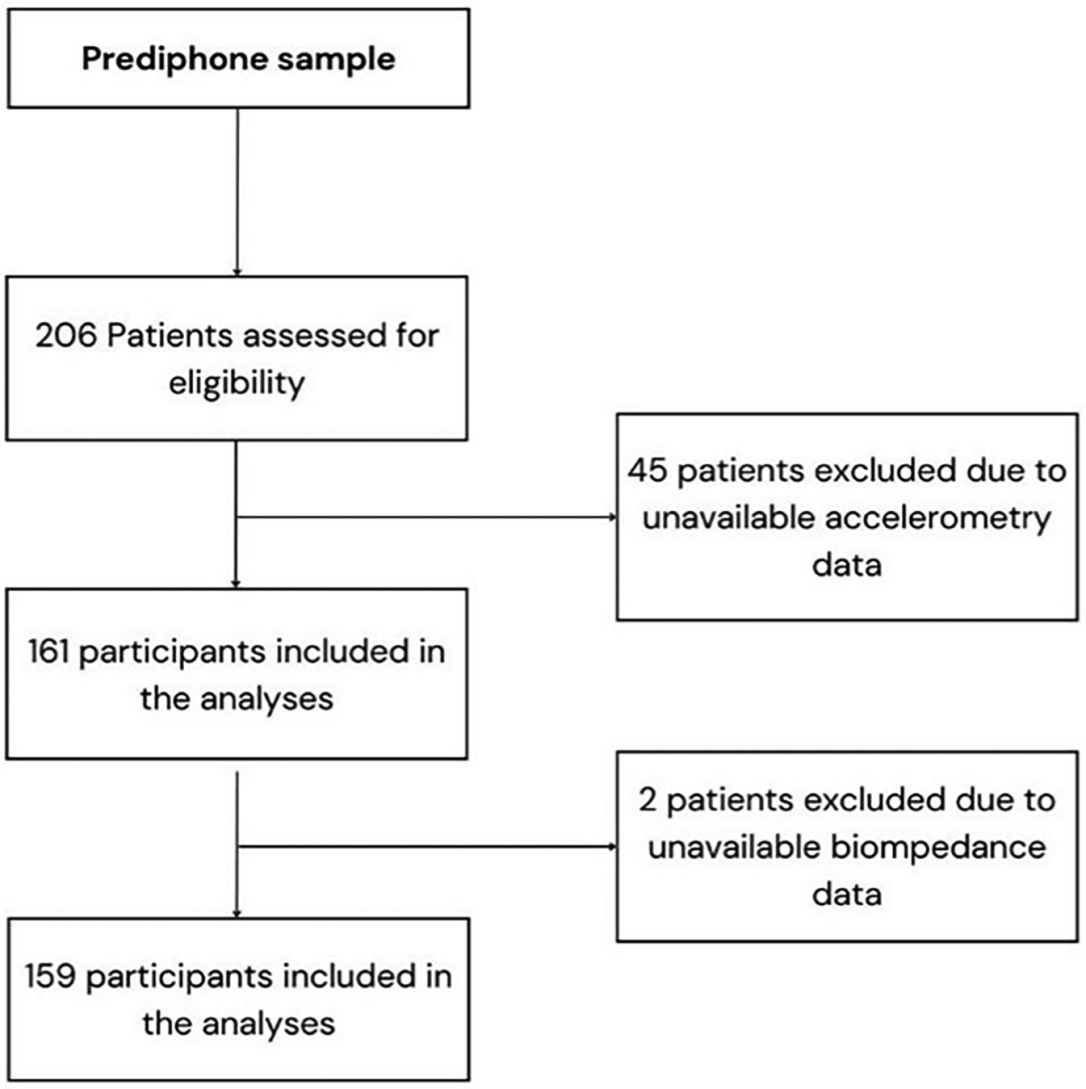
Flow chart of study sample selection from the prediphone study.

Of the 206 participants, 159 had valid data available for accelerometry and bioimpedance, and were included in the analysis. Figure 1 shows the flow chart for sample selection (32).

### Data collection and measurements

Data was collected at baseline visits by qualified healthcare professionals and included sociodemographic characteristics (age, sex, educational level, and occupation), medical history, use of medications, and smoking behavior.

Participants were categorized as *white collar* (executives, managers, university professionals, intermediate workers, and employees) or *blue collar* (manual laborers), according to the occupation they self-reported (33).

Smoking behavior was classified as *never*, *former*, or *current smoker* according to the World Health Organization (WHO) classification.

Anthropometric measurements were collected following the International Standards for Anthropometric Assessment (ISAK) (34). A body composition scale for body weight with bioelectrical impedance analysis (Tanita BC-418, Tanita Corp, Tokyo, Japan) was used to determine weight to the nearest 0.1 kg and body composition (35). Height was measured using a stadiometer [Seca 220 (CM) Telescopic Height Rod for Column Scales, Seca GmbH, Hamburg, Germany] with the participant standing straight and the head in the anatomical position. BMI was calculated using the standard formula [weight (kg)/height (m^2^)] that classifies obesity as BMI ≥ 30 kg/m^2^. Waist circumference (WC) was measured in double, halfway between the last rib and the top of the iliac crest, using an anthropometric tape, with participants standing upright, with arms folded across the chest and feet evenly spread apart. The mean of the two measurements was considered for statistical analyses. Waist circumference cut-off points for abdominal obesity were ≥88 cm for women and ≥102 cm for men (36).

Blood pressure (BP) was measured using an electric sphygmomanometer (OMRON M3, Healthcare Europe, Barcelona, Spain) with the participant sitting after 15 min of rest. The average of two measurements, one minute apart, was recorded (37). The arm used for BP reading was the one showing the highest BP.

Venous blood samples were collected from the antecubital vein in suitable vacutainers following an 8–12 h overnight fast.

Physical activity, sleeping time and ST were derived using an accelerometer (GENEActive, ActivInsights Ltd., Cambridgeshire, United Kingdom) on their non-dominant wrist, during 7 consecutive days after the baseline visit. The GENEActiv accelerometer collects triaxial (*x*, *y*, and *z*) accelerometer data to assess patterns of light, moderate, and vigorous PA, sedentary time and sleep (38). It has a dynamic range of ±8 *g*, which is equivalent to the Earth's gravitational force. It was programmed to collect and store accelerations with a sampling frequency of 40 Hz.

Data extracted from the GENEActiv (all in bouts of at least 1 min) were clustered as: inactive time (cut-off intensity level <40 mg) for those behaviors during waking hours equivalent to <1.5 Metabolic Equivalent Tasks (METs); light-intensity PA (LPA) (39) (cut-off intensity level between ≥40 mg and <100 mg) equivalent to 1.5–3 METs; moderate-to-vigorous PA (MVPA) (cut-off intensity level ≥100 mg) equivalent to >3 METs; and sleeping time (time between going to bed and getting up, calculated using a validated heuristic algorithm from accelerometer raw data unaided by a sleep diary) (40–42). MVPA was analyzed together, as this population practiced very (LPA. The University of the Balearic Islands maintained the raw data files on its servers. To process the data, the open-source R-package GGIR, version 4.2–1 (cran.rproject.org/web/packages/GGIR/index. html), was utilized. The package was run using R (R Core Team, Vienna, Austria). The open-source code in GGIR has been verified against functions that self-calibrate, ensuring its reliability (43).

### Statistical analysis

Categorical variables were expressed as numbers and percentages. Chi-square test (*χ*^2^) was used to assess the associations between categorical variables, such the association between as low or high VAT and social class and smoking habits. Continuous variables were summarized as means and standard deviations (SDs) and associations were evaluated using analysis of variance.

Linear regression analyses were used to estimate the β-coefficients and 95% CI for the associations between PA and ST with body composition, using accelerometry data and bioimpedance, respectively.

Isotemporal substitution linear regression modelling was performed to examine the associations of replacing ST with sleep time, LPA, or MVPA on body composition. The resulting regression coefficient represents the effect of reallocating a unit of ST to a unit of sleep time, LPA, or MVPA. The model 1 was adjusted for age and sex, and the model 2 was further adjusted for social class, smoking status and adherence to mediterranean diet. The analyses followed published guidelines for isotemporal substitution (29). The isotemporal substitution model allows for the replacement of behaviours while accounting for the finite nature of time (24 h per day). This approach aligns with individuals' daily realities, enabling a more accurate and realistic interpretation of the data, with greater applicability to recommendations on healthy habits.

A *P*-values < 0.05 were considered statistically significant. All analyses were conducted using Stata v.16 software (StataCorp, Texas, USA).

## Results

Of the 159 participants with prediabetes and overweight/obesity included in the present analysis, 52.8% were women. Mean age was 59.6 (10.1) years. Sociodemographic characteristics, smoking habits, anthropometric measures, body composition, clinical parameters, and 24 h movement behaviors of the sample as a whole and by VAT accumulation were shown in Table 1. Moreover, the same date has shown by sex in Supplementary Table S1. Most subjects (78%) were blue-collar and non-smokers (47.2%). The mean BMI was 32.1 [standard deviation (SD) 3.6]. Regarding body composition, the mean VAT was 13.1 (SD 3.9), and the mean body fat mass in percentage (%) was 35.8 (SD 7.0). The behavior that accumulated the most time in 24 h was ST, with an average of 751 (SD 93.4) minutes, or 12.5 h per day. Light PA was the main type of exercise performed, with a mean accumulation of181.1 min per day (SD 55.5), followed by MPA, with a mean of 108.4 min (SD 55.7). Participants only performed a mean of 2.7 (SD 3.6) minutes per day of VPA.

**Table 1:** Characteristics of the participants by VAT accumulation.

Variables	All (*n* = 159)	Low VAT (*n* = 34)	Moderate VAT (*n* = 70)	High VAT (*n* = 55)	*p*-value
Sociodemografic variables					
Age	59.6 (10.1)	54.8 (12.1)	60.3 (10.4)	61.8 (7.3)	0.005
Sex					<0.001
Woman [*n*, (%)]	84 (52.8)	31 (91.2)	48 (68.6)	5 (9.1)	
Men [*n*, (%)]	75 (47.2)	3 (8.8)	22 (31.4)	50 (90.9)	
Social class					0.130
White collar [*n*, (%)]	35 (22.0)	5 (14.7)	13 (18.6)	17 (30.9)	
Blue collar [*n*, (%)]	124 (78.0)	29 (85.3)	57 (81.4)	38 (69.1)	
Smoking habits					0.001
Never [*n*, (%)]	75 (47.2)	24 (70.6)	36 (51.4)	15 (27.3)	
Current [*n*, (%)]	21 (13.2)	5 (14.7)	8 (11.4)	8 (14.5)	
Former [*n*, (%)]	63 (39.6)	5 (14.7)	26 (37.1)	32 (58.2)	
Diet Adherence					0.577
High Adherence	72 (45.3)	14 (41.2)	30 (42.9)	28 (50.9)	
Low adherence	87 (54.7)	20 (58.8)	40 (57.1)	27 (49.1)	
Anthropometric measures					
BMI (kg/m^2^)	32.1 (3.6)	29.6 (2.6)	32.2 (3.4)	33.5 (3.7)	<0.001
WC (cm)	105.1 (10.5)	94.5 (8.3)	103.9 (7.6)	113.2 (8.1)	<0.001
Abdominal obesity^a^ [*n*, (%)]	136 (85.5)	26 (76.5)	58 (82.9)	52 (94.6)	0.043
Body composition					
VAT (units)	13.1 (3.9)	8.2 (0.9)	12.0 (1.3)	17.5 (2.5)	<0.001
Body fat mass (kg)	30.7 (8.0)	25.5 (4.5)	31.8 (8.3)	32.4 (8.0)	0.001
Body fat mass (%)	35.8 (7.0)	35.4 (5.0)	37.9 (8.2)	33.5 (5.5)	0.002
Lean body mass (kg)	54.9 (11.3)	46.7 (7.3)	52.0 (9.6)	63.8 (9.4)	<0.001
Clinical parameters					
SBP (mmHg)	134.9 (14.9)	126.9 (13.5)	134.2 (14.5)	140.8 (13.9)	0.001
DBP (mmHg)	84.0 (9.3)	78.6 (7.7)	84.0 (8.6)	87.4 (9.7)	0.001
HbA1c (%)	5.9 (0.3)	5.8 (0.3)	5.9 (0.3)	5.9 (0.4)	0.494
Total cholesterol (mg/dl)	201.5 (36.0)	205.8 (23.5)	204.4 (36.8)	195.5 (40.8)	0.300
HDL-C (mg/dl)	50.5 (12.5)	53.5 (9.3)	52.3 (14.9)	46.5 (10.0)	0.012
LDL-C (mg/dl)	121.9 (30.3)	125.7 (25.8)	124.1 (32.0)	117.1 (30.9)	0.344
Triglycerides (mg/dl)	156.5 (155.2)	127.9 (45.1)	153.2 (136.2)	171.6 (208.6)	0.340
24 h behaviours (min/d accelerometry)					
LPA	181.1 (55.5)	198.0 (53.7)	183.7 (55.4)	167.5 (54.2)	0.035
MPA	108.4 (55.7)	130.7 (61.5)	105.9 (53.4)	98.0 (52.0)	0.022
VPA	2.7 (3.6)	2.8 (2.8)	2.7 (4.3)	2.6 (3.2)	0.965
MVPA	111.1 (67.6)	133.5 (63.1)	108.6 (55.7)	100.5 (53.5)	0.028
ST	751.0 (93.4)	715.7 (95.3)	752.6 (87.9)	770.8 (94.5)	0.024
Sleep (min/d)	306.7 (52.4)	301.3 (54.7)	309.5 (49.4)	306.5 (55.5)	0.758
Sleep (h/d)	5.1 (0.9)	5.02 (0.8)	5.2 (1.0)	5.1 (0.9)	0.758

Data was expressed as mean (SD) and as count (percentage). VAT was defined low (0–9 units), moderate (10–14 units), and high (≥15 units). Adherence to Mediterranean Diet was classified as high (≥8 points) or low (≤7 points) in 14 items Mediterranean Diet Questionnaire.

BMI, body mass index; WC, waist circumference; VAT, visceral adipose tissue; SBP, systolic blood pressure; DBP, diastolic blood pressure; HbA1c, glycosylated haemoglobin; HDL, high-density lipoprotein; LDL, low-density lipoprotein; PA, physical activity; min/d-minutes per day; LPA, light-intensity physical activity; MPA, moderate physical activity; VPA, vigorous physical activity; ST, sedentary time; h/d, hours per day.

^a^ Abdominal obesity: waist circumference ≥88 cm for women and ≥102 cm for men.

As compared to women, men presented a significantly lower fat body mass (%), but higher VAT (all *p* < 0.001), although women were more often abdominally obese than men (*p* = 0.02). Men also presented higher systolic and diastolic BP (*p* = 0.005 and *p* = 0.01, respectively) and lower HDL-cholesterol levels (*p* < 0.001) when compared to women. No differences between sexes were observed in 24 h movement behaviors (see Supplementary Table S1).

Table 1 provides an overview of the study sample categorized according to VAT accumulation. The population was classified into three groups: “low VAT accumulation” (0–9 units based on tetrapolar bioimpedance) with a mean 8.2 (SD 0.9), “moderate VAT accumulation” (10–14 units) with a mean 12.0 (SD1.3), and “high VAT accumulation” (≥15 units) with a mean 17.5 (SD 2.5). The population with high VAT accumulation was older, predominantly male, were former smokers (less than two years without smoke), and has higher BMI, greater WC, and has abdominal obesity as measured by WC. All *p*-values were significant. No significant differences were observed in relation to social class or adherence to the Mediterranean diet. Regarding body composition, participants with high VAT accumulation had greater total fat mass (kg) and muscle mass (kg) but lower total fat percentage. All *p*-values were significant. Concerning clinical parameters, the population with higher VAT presents higher SBP and DBP and lower HDL-c levels. In terms of 24 h behaviours, participants with higher VAT engaged in less LPA, MPA, and VPA while spending more time in sedentary activities. All *p*-values were significant.

The associations between ST, PA and sleeping time with BMI, WC and body composition variables are shown in Table 2. As expected, ST was positively and significantly associated with body fat mass (kg) (*β* = 0.016; CI 95%: 0.003–0.030), body fat mass (%) (*β* = 0.009; CI 95%: 0.001–0.018) and VAT (*β* = 0.005; CI 95%: 0.001–0.010). Moderate-to-vigorous PA was negatively and significantly associated with body fat mass (kg) [*β* = −0.031; CI 95%: 0.055- (−0.008)], body fat mass (%) [*β* = −0.017; CI 95%: −0.032-(−0.003)] and VAT [*β* = −0.009; CI 95%: −0.02-(−0.002)]. No significant associations were observed between LPA or sleeping time with any body composition variables. Results were consistent between models 1 and 2. When further adjusting for Mediterranean diet, social class and smoking, sleep and sedentary time were not significantly associated with VAT nor body fat. The remaining associations did not change with the additional adjustments.

**Table 2:** Association of sedentary time, physical activity, and sleep with body composition.

Variable	ST (min/d)			LPA (min/d)			MVPA (min/d)			Sleep (min/d)		
	β	95% CI	*p*	β	95% CI	*p*	β	95% CI	*p*	β	95% CI	*p*
Anthropometric measures												
BMI												
Model 1	0.004	−0.003; 0.010	0.259	−0.002	0.013; 0.008	*0* *.* *657*	−0.009	−0.021; 0.002	*0* *.* *101*	−0.002	0.013; 0.009	0.739
Model 2	0.003	−0.004; 0.010	0.351	−0.001	−0.012; 0.010	*0* *.* *856*	−0.009	−0.020; 0.003	*0* *.* *128*	−0.002	−0.013; 0.009	0.731
WC												
Model 1	0.022	0.005; 0.039	0.014	−0.029	−0.057; 0.001	*0* *.* *051*	−0.038	−0.068; −0.007	*0* *.* *016*	0.002	−0.029; 0.032	0.912
Model 2	0.019	0.002; 0.037	0.034	−0.022	−0.052; 0.007	*0* *.* *136*	−0.034	−0.065; −0.004	*0* *.* *027*	−0.001	−0.032; 0.030	0.956
Body composition												
VAT^a^ (units)												
Model 1	0.005	0.001; 0.010	0.023	−0.006	−0.014; 0.001	0.103	−0.009	−0.02; −0.002	0.020	0.003	−0.005; 0.011	0.461
Model 2	0.004	−0.001; 0.009	0.078	−0.004	−0.012; 0.004	0.332	−0.009	−0.017; −0.001	0.041	0.002	−0.006; 0.010	0.553
Body fat mass^a^ (kg)												
Model 1	0.016	0.003; 0.030	0.019	−0.018	−0.041; 0.004	0.109	−0.031	−0.055; −0.008	0.009	0.005	−0.018; 0.029	0.652
Model 2	0.013	−0.001; 0.027	0.067	−0.010	−0.032; 0.013	0.394	−0.030	−0.051; −0.004	0.020	0.001	−0.023; 0.025	0.946
Body fat mass^a^ (%)												
Model 1	0.009	0.001; 0.018	0.025	−0.013	−0.027; 0.000	0.058	−0.017	−0.032; −0.003	0.020	0.010	−0.004; 0.025	0.171
Model 2	0.008	−0.001; 0.016	0.080	0.009	−0.023; 0.005	0.204	−0.015	−0.030; −0.001	0.041	0.009	−0.006; 0.024	0.231
Lean body mass^a^ (kg)												
Model 1	0.005	−0.007–0.017	0.406	0.003	−0.018–0.023	0.795	−0.011	−0.032–0.011	0.318	−0.019	−0.041–0.001	0.064
Model 2	0.004	−0.009; 0.016	0.569	0.007	−0.013; 0.028	0.481	−0.009	−0.031; 0.013	0.402	−0.025	−0.047; −0.004	0.021

Linear regression models were used to assess the association between PA, ST and body composition outcomes. Model 1 adjusted sex, and age. Model 2 further adjusted for adherence to mediterranean diet, social class and smoking habits. Lean mass analyses were further adjusted for weight.

ST, sedentary time; min/d, minutes per day; LPA, light-intensity physical activity; MVPA, moderate-to-vigorous physical activity; BMI, body mass index; WC, waist circumference; VAT, visceral adipose tissue; β-regression coefficient; CI, confidence interval.

^a^ Model 1 adjusted sex, and age. Model 2 further adjusted for adherence to mediterranean diet, social class and smoking habits.

The β-coefficients (95% CI) of the isotemporal substitution models are shown in Table 3. Isotemporal substitution of 1 min per day of ST with the equivalent time of MVPA (i.e., decreasing ST at the expenses of increasing MVPA time, without changing the time spent on other behaviors) was associated with less VAT [*β* = −0.012; CI 95%: −0.024-(−0.001)] and less body fat mass (kg) [*β* = −0.039; CI 95%: −0.074-(−0.006)]. Reallocating 1 min of ST for LPA or sleeping time was not associated with any of the body composition variables, nor WC or BMI. There were trends indicating associations between increased levels of any PA and greater lean mass, as well as replacing ST with sleeping time and lower lean mass but did not reach statistical significance. The results obtained for Model 1 and Model 2 were consistent and exhibited minimal variation.

**Table 3:** Isotemporal substitution of sitting time with physical activity and sleep time on body composition and anthropometric measures.

Isotemporal substitution	ST with sleep			ST with LPA			ST with MVPA		
	β	95% CI	*p*	β	95% CI	*p*	β	95% CI	*p*
Outcome				Anthropometric measures					
BMI (kg/m^2^)									
Model 1	−0.002	−0.022;0.017	0.831	−0.002	−0.008;0.020	0.400	−0.014	−0.030;0.001	0.083
Model 2	−0.002	−0.023; 0.019	0.852	0.008	−0.007; 0.024	0.279	−0.015	−0.031; 0.001	0.070
WC (cm)									
Model 1	−0.019	−0.071;0.033	0.473	−0.001	−0.051;0.029	0.588	−0.035	−0.079;0.009	0.117
Model 2	−0.020	−0.076;0.035	0.477	−0.002	−0.041; 0.039	0.918	−0.038	−0.082; 0.005	0.085
Body composition									
VAT (units)									
Model 1	−0.007	−0.022; 0.007	0.327	−0.001	−0.011;0.010	0.944	−0.012	−0.024; −0.001	0.041
Model 2	−0.006	−0.021; 0.008	0.397	0.003	−0.008; 0.014	0.600	−0.013	−0.025; −0.001	0.028
Body fat mass (kg)									
Model 1	−0.016	−0.059;0.025	0.435	0.003	−0.028;0.034	0.848	−0.039	−0.074; −0.006	0.022
Model 2	−0.016	−0.058; 0.027	0.466	0.014	−0.017; 0.045	0.376	−0.043	−0.076; −0.009	0.012
Body fat mass (%)									
Model 1	−0.006	−0.033;0.019	0.619	−0.003	−0.022;0.015	0.748	−0.019	−0.039;0.002	0.076
Model 2	−0.005	−0.031; 0.022	0.721	0.002	−0.017; 0.022	0.817	−0.020	−0.041; 0.001	0.057
Lean body mass (kg)									
Model 1	−0.007	−0.029; 0.014	0.497	0.007	−0.008;0.023	0.363	0.003	−0.015;0.020	0.761
Model 2	−0.010	−0.032;0.012	0.384	0.005	−0.011; 0.022	0.536	0.003	−0.015; 0.021	0.734

Isotemporal substitution analyses were used to assess the replacement of ST with sleep, LPA and MVPA and body composition outcomes. Model 1 adjusted sex, and age. Model 2 further adjusted for adherence to mediterranean diet, social class and smoking habits. Lean mass analyses were further adjusted for weight. Total sample size was *n* = 159.

ST, sedentary time; LPA, light-intensity physical activity; MVPA, moderate-to-vigorous physical activity; β, regression coefficient; CI, confidence interval; BMI, body mass index; WC, waist circumference; VAT, visceral adipose tissue.

## Discussion

The main results from the present cross-sectional analysis including middle-aged individuals with prediabetes and overweight or obesity show that an increase in ST was associated with increased VAT and body fat mass, while an increase in MVPA was associated with reduced VAT and body fat mass. Moreover, replacing ST with MVPA was associated with lower VAT and body fat mass accumulation, but not with changes in lean mass. Of note, only a small proportion of individuals regularly engaged in MVPA, with the vast majority engaging in none or LPA, which is characteristic of the obese population (44).

Our findings are in line with previous evidence showing that when ST is replaced with MVPA it is associated with improved body composition and specifically less total body fat and VAT (45, 46).

We did not find associations between replacing ST with LPA and changes in body composition. This is in contrast with other studies that have shown the potential beneficial effect of substituting ST with LPA on health outcomes in high-risk populations with prediabetes and T2D (47). Previous research has in fact showed a significant decrease in fat mass (48) and VAT associated to LPA increase in older population with overweight or obesity (45). However, these effects were less pronounced compared to substituting ST with MVPA, possibly indicating that the degree of body and visceral fat loss is dependent on the intensity of the PA.

Replacing ST with sleeping time did not improve weight or body composition in our sample. This is also in contrast with other studies that found this substitution associated with lower BMI and WC (45). Sleep loss is linked to increased inflammation and hormonal disruptions, including insulin, leptin and ghrelin, which can affect energy balance and body weight regulation (49). However, it has also been observed that disturbed sleep rather than sleep loss can increase cardiometabolic risk factors, including weight gain, in the long term (50, 51). The lack of association in our study may be due to the fact that we considered sleep duration rather than sleep quality, possibly masking the potential benefits of replacing ST with sleeping time if the quality of sleep was poor.

Additionally, our results did not show associations between any PA level and lean mass, and a non-significant trend indicating associations between increased levels of any PA and greater lean mass. Previous studies show that engaging in MVPA and reducing ST were associated with a higher lean mass (52). Even among older people, increased time dedicated to MVPA, and reduced ST were linked to improved body composition and a decreased likelihood of loss of muscle mass (53). The discrepancies with our findings may be explained by the fact that lean mass, like fat mass, is dependent of PA intensity. In our sample, the main type of PA performed for MVPA was moderate rather than vigorous (98.1% of women and 96.7% of men performed MPA within the MVPA category), and thus not enough intensive to significantly impact lean mass. Previous studies, in fact, observe that achieving a significant change in lean mass may require more intensive PA (45, 53, 54). Since skeletal muscle accounts for 80% of insulin-stimulated glucose disposal, and that obesity is associated with increasing insulin resistance in muscle tissue (55, 56), maintaining muscle mass while reducing total fat and VAT should always be advised when loss of weight is recommended.

Importantly, the dose-dependent effect of PA is not limited to body composition. Although it has been observed that any PA intensity is beneficial for overall health and chronic disease prevention (54), including CVD and T2D (47, 57, 58), VPA shows the highest risk reduction (47), making it one of the main strategies to maintain and improve individual's health.

A marked strength of this study was the objective and validated measurements used for both exposure and outcome variables, increasing the opportunities for comparison across the literature and reducing any potential bias or measurement error. Both the tetra-polar bioimpedance and accelerometry are valid, quick, and easy-to-use techniques (35, 38). The accelerometry electronic devices record the acceleration linked to body movements, offering an objective estimate of the movement's duration and intensity (45, 54). Additionally, the isotemporal substitution analysis represents a robust and novel research methodology.

In terms of limitations, the cross-sectional design limits drawing conclusions on causality or evaluating changes in body composition over the time. Our findings suggest associations between the ST, PA, and body composition, but the nature of these relationships remains observational. Previous longitudinal and intervention studies indicate that decreasing sedentary time (ST) and increasing physical activity (PA) may contribute to enhancements in body composition (59–62), but our results should be interpreted as correlational rather than causal. Future prospective and experimental studies are needed to confirm these associations and better understand the direction of effect. Secondly, the modest sample size may limit the generalizability of the results to individuals with prediabetes and overweight or obese.

In conclusion, this study showed that among middle-aged individuals with prediabetes and overweight or obesity, increased ST is associated with higher VAT and body fat mass, while MVPA is associated with reductions in both. Substituting ST with MVPA could lower VAT and body fat mass but does not increase lean mass. Rather than solely focusing on body weight, weight loss recommendations should be aimed at reducing adipose tissue while preserving or improving muscle mass.

## Data Availability

The raw data supporting the conclusions of this article will be made available by the authors, without undue reservation.
