# Supplementary material for: Isotemporal substitution of sedentary time with physical activity and sleeping time: associations with body composition among individuals with prediabetes
Source: Front Sports Act Living. 2025 Apr 7;7:1579962. doi: 10.3389/fspor.2025.1579962 (PMC12009759; doi:10.3389/fspor.2025.1579962)
Supplement: Supplementary file 1 [file Table1.docx]

**Supplementary Table 1. Characteristics of the participants by sex.**

| **Variables** | **All (n=159)** | **Women n=84** | **Men n=75** | **p-value** |
| --- | --- | --- | --- | --- |
| **Sociodemografic variables** | | | | |
| Age | 59.6 (10.1) | 60.3 (10.1) | 58.9 (10.2) | 0.373 |
| Social class |  |  |  | 0.568 |
| White collar (n, (%)) | 35 (22.0) | 17 (20.2) | 18 (24.0) |  |
| Blue collar (n, (%)) | 124 (78.0) | 67 (79.8) | 57 (76.0) |  |
| Smoking habits |  |  |  | <0.001 |
| Never (n, (%)) | 75 (47.2) | 53 (63.1) | 22 (29.3) |  |
| Current (n, (%)) | 21 (13.2) | 13 (15.5) | 8 (10.7) |  |
| Former (n, (%)) | 63 (39.6) | 18 (21.4) | 45 (60.0) |  |
| **Anthropometric measures** | | | | |
| BMI (kg/m^2^) | 32.1 (3.6) | 32.3 (3.8) | 31.8 (3.4) | 0.380 |
| WC (cm) | 105.1 (10.5) | 101.9 (10.5) | 108.6 (9.2) | <0.001 |
| Abdominal obesity* (n, (%)) | 136 (85.5) | 77 (91.7) | 59 (78.7) | 0.020 |
| **Body composition** | | | | |
| VAT (units) | 13.1 (3.9) | 10.6 (2.4) | 15.8 (3.5) | <0.001 |
| Body fat mass (kg) | 30.7 (8.0) | 32.7 (7.9) | 28.4 (7.5) | 0.001 |
| Body fat mass (%) | 35.8 (7.0) | 40.6 (4.8) | 30.4 (4.9) | <0.001 |
| Lean body mass (kg) | 54.9 (11.3) | 48.9 (5.74) | 64.0 (8.9) | <0.001 |
| **Clinical parameters** | | | | |
| SBP (mmHg) | 134.9 (14.9) | 131.8 (15.0) | 138.4 (14.0) | 0.005 |
| DBP (mmHg) | 84.0 (9.3) | 82.2 (9.1) | 86.0 (9.2) | 0.010 |
| HbA1c (%) | 5.9 (0.3) | 5.9 (0.3) | 5.9 (0.3) | 0.742 |
| Total cholesterol (mg/dl) | 201.5 (36.0) | 206.3 (31.1) | 196.3 (40.2) | 0.086 |
| HDL-C (mg/dl) | 50.5 (12.5) | 54.5 (13.7) | 46.1 (9.3) | <0.001 |
| LDL-C (mg/dl) | 121.9 (30.3) | 123.4 (28.8) | 120.4 (32.1) | 0.552 |
| Triglycerides (mg/dl) | 156.5 (155.2) | 144.4 (67.5) | 169.7 (212.6) | 0.318 |
| **24-h behaviours (min/d accelerometry)** | | | | |
| LPA | 181.1 (55.5) | 189.0 (54.7) | 172.3 (55.3) | 0.057 |
| MPA | 108.4 (55.7) | 105.9 (58.2) | 111.2 (53.0) | 0.549 |
| VPA | 2.7 (3.6) | 1.8 (2.1) | 3.7 (4.6) | 0.001 |
| MVPA | 111.1 (67.6) | 107.7 (59.6) | 114.9 (55.4) | 0.429 |
| ST | 751.0 (93.4) | 745.9 (95.4) | 756.7 (91.5) | 0.468 |
| Sleep | 306.7 (52.4) | 312.8 (48.1) | 299.8 (56.4) | 0.117 |
| Sleep (h/d) | 5.1 (0.9) | 5.2 (0.8) | 5.0 (0.9) | 0.117 |

Data was expressed as mean (SD) and as count (percentage). *Abdominal obesity: waist circumference ≥88cm for women and ≥102cm for men. *Abbreviations: BMI-Body Mass Index; WC-Waist circumference; VAT- Visceral adipose tissue; SBP-systolic blood pressure; DBP-diastolic blood pressure; HbA1c-glycosylated haemoglobin; HDL-High-density lipoprotein; LDL-Low-density lipoprotein; PA-Physical activity; min/d-minutes per day; LPA-Light-intensity physical activity; MPA-Moderate physical activity; VPA-Vigorous physical activity; ST-Sedentary time; h/d-hours per day.*
